# Mycetoma managment: Therapeutic challenges and the role of pharmacovigilance

**DOI:** 10.1371/journal.pntd.0012827

**Published:** 2025-02-20

**Authors:** Samira M. E. Hussein, Ali Awadallah Saeed, Ahmed Hassan Fahal

**Affiliations:** Mycetoma Research Center, Soba University Hospital, University of Khartoum, Khartoum, Sudan; Albert Einstein College of Medicine, UNITED STATES OF AMERICA

## Abstract

Mycetoma presents numerous therapeutic challenges, particularly due to delays in diagnosis, the toxic nature of existing antifungals and antibiotics treatments, and the lack of robust clinical evidence to guide care. This neglected tropical disease, which primarily affects low-resource regions, is further complicated by socio-economic barriers that limit access to healthcare and treatment. These challenges underscore the urgent need for better treatment options, more comprehensive research, and strengthened pharmacovigilance systems to monitor treatment safety and efficacy. Pharmacovigilance plays a critical role in managing mycetoma due to the prolonged and often toxic treatment regimens required. Adverse drug reactions, drug interactions, and treatment side effects need continuous monitoring to ensure patient safety. Effective pharmacovigilance systems should be adapted to the resource-limited settings where mycetoma is most prevalent, integrating into broader public health efforts to improve both the safety and efficacy of treatments. Such systems could greatly enhance patient outcomes by preventing unnecessary harm from toxic therapies and ensuring proper drug use. However, several barriers remain in endemic regions, including inadequate healthcare infrastructure, a lack of trained healthcare professionals, and limited access to pharmacovigilance tools. Addressing these issues requires building stronger national systems, offering more training for healthcare workers, and leveraging innovative technologies such as mobile health tools. Additionally, involving patients in reporting adverse effects could enhance the accuracy and reliability of pharmacovigilance data. Global collaboration and increased clinical research are also essential in improving mycetoma treatment. Investment in these areas, alongside the development of infrastructure and education in endemic countries, will help ensure safer long-term medical therapies and better outcomes for mycetoma patients. Furthermore, improving pharmacovigilance practices is critical to ensuring that vulnerable populations receive the most effective and safe care possible for this neglected disease.

## Background

Mycetoma is a chronic, progressively destructive granulomatous inflammatory disease caused by true fungi and actinomycetes, leading to eumycetoma and actinomycetoma, respectively [1,2]. This debilitating disease predominantly afflicts impoverished populations residing in tropical and subtropical regions, where access to healthcare is often limited [3–5]. The progression of mycetoma is typically slow but relentless, frequently leading to severe physical disability, disfigurement, and social stigma, contributing to the isolation of affected individuals [6,7]. Despite its long history and significant impact on patients and communities, mycetoma was only recognised by the World Health Organization (WHO) as a neglected tropical disease (NTD) in 2016. This late recognition has led to increased attention, but therapeutic options remain scarce [8].

The mainstay of treatment involves long-term administration of antifungal agents or antibiotics [9–12]. However, these medications are often associated with serious side effects, including hepatotoxicity, adrenal and renal impairment and cutaneous and gastrointestinal disturbances, which can be exacerbated by the extended duration of therapy required to control the infection [10–12]. This presents a significant challenge in managing the disease, as the risks associated with prolonged drug use often limit treatment adherence and success [10].

Given the chronic nature of mycetoma and the reliance on potentially toxic drugs [13], pharmacovigilance becomes crucial in ensuring the safety of patients. Pharmacovigilance is defined by the WHO as the science and activities relating to the detection, assessment, understanding, and prevention of adverse effects or any other medicine/vaccine-related problem [13]. Pharmacovigilance is essential for monitoring adverse drug reactions (ADRs), optimising treatment protocols, and safeguarding patient health by detecting and preventing harmful drug-related events [13].

This communication will discuss the integral relationship between pharmacovigilance and mycetoma management, emphasising the critical role that drug safety plays in addressing this prevalent but neglected global health issue. It aims to explore how pharmacovigilance can mitigate risks, enhance patient outcomes, and guide future therapeutic strategies in treating mycetoma, as well as explore the Mycetoma Research Center’s experience in ensuring patient safety.

## Mycetoma: a complex therapeutic challenge

Managing mycetoma presents several encounters, including delayed diagnosis and misdiagnosis, limited access to diagnostic tools, prolonged treatment duration, drug toxicity and adverse effects, and psychosocial and economic impact. All these are due to the disease nature, the limitations of available therapies, and the socio-economic context in which it primarily occurs [1,3,4,10–12]. Several factors contribute to the complexity of mycetoma management, including:

### 1. Difficulty in early diagnosis

One of the primary challenges in treating mycetoma is the difficulty of early diagnosis [9]. It typically starts as a painless subcutaneous swelling, which may be misdiagnosed or overlooked by both patients and healthcare providers. Mycetoma enjoys all the other characteristics and challenges of skin-neglected tropical diseases [1,3,7]. Hence, the disease may be mistaken for other tropical infections or conditions, delaying appropriate diagnosis and treatment [2,9,14]. By the time it is accurately identified, the infection has frequently advanced to later stages, involving deeper tissues and bone [2,9,14]. This delay complicates treatment, as advanced mycetoma often requires more aggressive and prolonged interventions, including massive surgical excisions or even amputation in severe cases [2,14–17].

### 2. Long-term and toxic antifungal treatments

For eumycetoma, antifungal agents such as itraconazole and ketoconazole are the treatment cornerstone [2,11,15]. However, these therapies require extended courses, often lasting months or even years, which poses significant risks to patients. Long-term use of these drugs is associated with considerable side effects [11,12].

Itraconazole can lead to hepatotoxicity, necessitating regular liver function monitoring throughout the treatment period [11,18,19]. Ketoconazole is linked to severe adrenal and hepatic side effects, and its systemic use has been restricted in many countries due to safety concerns [11,20,21]. Terbinafine is less frequently used in the treatment of mycetoma due to its limited efficacy against fungi that cause eumycetoma, poor tissue penetration, and insufficient clinical evidence supporting its effectiveness. Although it is generally well-tolerated, it may still cause side effects such as gastrointestinal disturbances, skin reactions, and, in rare instances, liver damage [22,23,24].

### 3. Actinomycetoma treatment

It typically involves prolonged use of antibiotics such as amikacin sulphate, streptomycin, cotrimoxazole, augmentin, dapsone and others, often combined for months or even years [9,12]. While these drugs are effective, they can cause significant toxicity and side effects. Amikacin sulphate and streptomycin are known to cause nephrotoxicity and ototoxicity, which may be irreversible with long-term use [25]. Cotrimoxazole can lead to severe skin reactions, bone marrow suppression, and liver toxicity [26]. Dapsone, though generally well-tolerated, may cause haemolysis, and methemoglobinemia, leading to cyanosis [27]. The prolonged and high-dose nature of treatment increases the risk of these adverse effects, necessitating regular monitoring. All these side effects can severely limit patient adherence, particularly in resource-limited settings where routine monitoring and supportive care may be inaccessible. Furthermore, patients with comorbidities, such as malnutrition or liver disease, are at even greater risk of experiencing drug-related complications.

### 4. Lack of Robust clinical trials and evidence

Another significant hurdle in mycetoma treatment is the paucity of well-conducted, large-scale clinical trials that evaluate the efficacy and safety of current therapies [10,11]. Most treatment regimens are based on limited studies, case reports, or anecdotal evidence, leading to uncertainty about the optimal management of the disease [1,8,10,11]. The absence of randomised clinical trials (RCTs) has hindered the development of standardised treatment protocols, making it challenging for healthcare providers to determine the best therapeutic approach for individual patients.

Additionally, the efficacy of the different antifungal agents against eumycetoma is inconsistent, with cure rates varying widely depending on the strain of the causative organism, the extent of the disease, and patient adherence [10]. The lack of reliable data on drug efficacy further complicates treatment decisions and underscores the need for more research into mycetoma-specific therapies.

### 5. Limited pharmaceutical pipeline for new treatments

Despite its devastating impact, mycetoma remains an orphan disease with little attention from the pharmaceutical industry. There are currently no novel antifungal drugs specifically developed for mycetoma in the pharmaceutical pipeline [10]. This lack of innovation is largely due to the disease status as a neglected tropical disease, which predominantly affects low-income populations in regions with limited commercial appeal for drug development [1,8,28].

Without the development of new, more effective, and safer treatment options, healthcare providers must rely on existing medications that are often poorly tolerated and insufficiently effective [10,29]. This emphasises the need for pharmacovigilance to closely monitor the safety profiles of these drugs and identify potential risks that may arise during long-term treatment.

### 6. Socio-economic barriers to treatment

Compounding the clinical challenges of mycetoma are the socio-economic barriers faced by patients in endemic areas. Mycetoma primarily affects people in low-income, rural communities where access to healthcare services is limited [30–33]. Patients often lack the financial resources to afford long-term antifungal therapy, which can be prohibitively expensive [30–33]. Additionally, healthcare facilities in these regions may not have the infrastructure needed to monitor patients for drug toxicity, conduct laboratory tests, or provide comprehensive care, further complicating treatment efforts [10].

### The role of pharmacovigilance in mycetoma management

Pharmacovigilance, the science of detecting, assessing, understanding, and preventing adverse effects or any other drug-related issues, is essential for the safe and effective management of mycetoma [13,34]. Given the chronic and often long-term nature of treatment for mycetoma, the likelihood of adverse drug reactions (ADRs) is significant. Effective pharmacovigilance systems are crucial for minimising the risks associated with prolonged medication use while optimising therapeutic benefits, especially in resource-limited settings where access to healthcare is already limited [34,35]. This is particularly important because many of the affected regions lack the infrastructure to monitor drug safety adequately. The roles of the pharmacovigilance in the management of mycetoma include:

#### 1. Monitoring adverse drug reactions (ADRs).

Antifungal agents such as itraconazole and ketoconazole are commonly used in treating eumycetoma, yet they are associated with a high risk of adverse effects. Given the long duration of antifungal therapy, which can last for months or even years, patients undergoing treatment must be closely monitored. [11,18,20,21] Pharmacovigilance systems provide the necessary framework to track these ADRs, enabling early detection of liver dysfunction or other side effects.

Regular liver function tests (LFTs) are essential for patients on antifungal medications to prevent irreversible liver damage [11,18,20,21]. Pharmacovigilance systems support this by ensuring that healthcare providers are alerted to potential hepatotoxicity early, prompting dose adjustments or drug substitutions when necessary. Additionally, these systems help document and analyse ADRs on a larger scale, which can inform future guidelines and improve treatment protocols for mycetoma.

Other common side effects from long-term antifungal use include gastrointestinal disturbances, skin reactions, and endocrine disruptions, particularly with ketoconazole [18,21]. Amikacin sulphate, commonly used for actinomycetoma treatment, is known for its nephro-ototoxicity, and hence, monitoring the renal and auditory functions closely is vital to preventing these serious complications [24,25].

By systematically documenting these reactions, pharmacovigilance systems create a more comprehensive understanding of the risks involved, ensuring that patients are monitored and managed appropriately.

#### 2. Drug interactions.

Patients with mycetoma, especially in endemic areas, often suffer from other comorbidities, such as malnutrition, tuberculosis, or diabetes, requiring the concurrent use of multiple medications [17]. Antifungal agents, particularly azoles like itraconazole and ketoconazole, are known to interact with various other drugs, complicating treatment regimens. For instance, itraconazole inhibits cytochrome P450 enzymes, which are responsible for metabolising many drugs, leading to the risk of increased toxicity when co-administered with medications like antiretrovirals or immunosuppressants metabolised by the same pathway [11,18,35,36].

Pharmacovigilance systems play a critical role in identifying and managing these drug-drug interactions (DDIs). Through continuous monitoring, pharmacovigilance can highlight potential interactions early, allowing healthcare providers to adjust dosing, switch to alternative therapies, or schedule treatments in a way that minimises interaction risks. These systems also provide valuable data for developing guidelines on managing comorbid conditions alongside mycetoma, thus improving overall patient safety and treatment outcomes.

Additionally, pharmacovigilance can track drug-food interactions, which can also affect the efficacy of antifungal therapy. For instance, the bioavailability of itraconazole increases when taken with food intake [37,38]. Unlike the capsules, itraconazole solution should be taken on an empty stomach for optimal absorption. The solution is absorbed better in the absence of food, as food can interfere with its absorption, reducing its bioavailability [39]. Itraconazole and ketoconazole are best absorbed under acidic conditions, potentially complicating treatment in patients who are also taking antacids or proton pump inhibitors [37,38].

Traditional remedies for mycetoma encompass a variety of herbal preparations, animal-derived products, and other natural substances, often sourced from local flora and fauna and can be tracked by pharmacovigilance. These treatments have been passed down through generations, with many patients seeking them as the first line of defence against the disease [40].

#### 3. Establishing Robust reporting systems.

In mycetoma-endemic regions, the lack of reliable and efficient pharmacovigilance reporting systems poses a significant challenge to patient safety. Establishing such systems is vital to ensuring that healthcare professionals can report ADRs, drug interactions, and other safety concerns promptly and effectively. This process involves creating accessible platforms for healthcare providers at all levels, including those working in rural or resource-poor settings, where most mycetoma patients reside [40].

Developing user-friendly reporting tools, such as mobile apps or SMS-based systems, can empower frontline healthcare workers to contribute to pharmacovigilance efforts, even with limited technological infrastructure. By streamlining the reporting process, healthcare professionals can quickly document ADRs, which can then be fed into national and international pharmacovigilance databases, such as the WHO’s global pharmacovigilance network [41].

Collecting data through these systems allows for the detection of patterns in adverse reactions, which can guide the development of safer treatment protocols. Moreover, these databases can help flag emerging safety concerns, such as resistance to antifungal drugs or regional variations in drug tolerance, prompting more localised or individualised approaches to mycetoma management [41].

#### 4. Adapting pharmacovigilance for resource-limited settings.

In low-and middle-income countries, where mycetoma is most prevalent, resource limitations often impede the effective implementation of pharmacovigilance activities. Healthcare systems in these regions may struggle with insufficient infrastructure, undertrained personnel, and a lack of financial resources to conduct regular monitoring of patients or maintain sophisticated data systems [35].

To overcome these barriers, pharmacovigilance initiatives must be adapted to fit local contexts. Simplified reporting systems, mobile health (mHealth) solutions, and community-based reporting networks can all play a role in ensuring that ADRs and drug interactions are captured, even in resource-constrained environments. Training healthcare workers in pharmacovigilance practices is equally critical to building local capacity for drug safety monitoring [41,42].

Furthermore, collaboration between national health ministries, international organisations, and non-governmental organisations (NGOs) can support the establishment of sustainable pharmacovigilance programmes in these regions. Such collaborations can also provide technical assistance, funding, and resources to ensure that pharmacovigilance efforts are integrated into routine healthcare services [41].

## The importance of patient-centered pharmacovigilance

In the management of mycetoma, one of the most critical aspects of pharmacovigilance is involving patients directly in the monitoring and reporting of drug safety. Mycetoma treatment is often lengthy, lasting several months to years, requiring strict adherence to medication regimens that can have significant adverse effects. Engaging patients in the pharmacovigilance process by educating them about the potential side effects and the importance of reporting any symptoms or concerns early is essential for both improving treatment outcomes and ensuring drug safety [42–44].

By educating patients on how to recognise the early signs of adverse drug reactions (ADRs), healthcare providers empower patients to become active participants in their care. This patient-centered approach not only improves treatment adherence, as patients better understand the necessity of completing their medication regimen but also enables the early identification of ADRs, which is critical for preventing more severe complications. For instance, patients can be taught to recognise symptoms of hepatotoxicity, such as jaundice or unusual fatigue, which are associated with antifungal therapies like itraconazole or ketoconazole. Early reporting of these symptoms allows for timely medical intervention, possibly preventing long-term damage.

Additionally, simplified reporting tools such as mobile phone apps, SMS-based systems, or toll-free hotlines can be invaluable in resource-limited settings, particularly in rural areas where healthcare access is scarce [45,46]. These tools provide an accessible and user-friendly way for patients to report any side effects or concerns directly to healthcare professionals. By creating direct communication channels, these systems enhance pharmacovigilance efforts, allow for real-time monitoring of patient safety, and help bridge the gap between patients and healthcare providers. Importantly, this participatory approach helps build trust between healthcare professionals and patients, a vital factor in resource-constrained environments where the healthcare system may already be strained [45].

### Pharmacovigilance challenges in mycetoma-endemic regions

Despite the critical importance of pharmacovigilance in managing mycetoma, several challenges make effective implementation difficult, particularly in regions where mycetoma is endemic. These challenges must be addressed to ensure the safe and effective use of long-term antifungal and antibacterial therapies.

#### 1. Lack of infrastructure.

In many mycetoma-endemic countries, healthcare infrastructure is inadequate, particularly in rural and underserved areas where the disease is most prevalent. Rural health centers often lack the necessary tools, technologies, and systems to monitor patients for ADRs consistently or to report these reactions to centralised pharmacovigilance databases. For example, regular hearing testing, critical for patients on amikicin treatments, may not be available in these areas. This gap in infrastructure hinders the timely identification of adverse drug reactions, putting patients at greater risk of drug-related complications. In addition to insufficient healthcare facilities, many regions also suffer from logistical challenges, including poor transport networks, making it difficult for patients to access medical care regularly. This can delay both the diagnosis of ADRs and the adjustment of treatment protocols, further complicating the already difficult task of managing a chronic disease like mycetoma.

#### 2. Inadequate training.

Pharmacovigilance activities in mycetoma-endemic regions are also often hampered by a lack of adequate training among healthcare professionals. Many healthcare workers in these regions may not be fully trained in the principles of pharmacovigilance, leading to underreporting of adverse drug reactions or failure to recognise early warning signs. Moreover, in resource-constrained healthcare systems that are overburdened by a high prevalence of diseases such as malaria and HIV, pharmacovigilance activities may be deprioritised in favour of more immediate healthcare needs.

Improving pharmacovigilance in these settings requires capacity building through specialised training programmes that educate healthcare workers on how to detect and report ADRs efficiently [47,48]. This could include workshops, online training modules, or on-site education that highlights the importance of pharmacovigilance, how to properly report drug-related problems, and how to use any available reporting tools. Building such capacity is key to creating a sustainable pharmacovigilance culture that can safeguard patient safety in the long term.

#### 3. Limited access to pharmacovigilance tools.

The absence of digital platforms and electronic health records (EHRs) in many mycetoma-endemic regions complicates the systematic collection, analysis, and reporting of pharmacovigilance data. Many healthcare providers still rely on paper-based systems, which are often inefficient and prone to errors, making it difficult to track ADRs over time or to detect patterns of drug-related harm. Furthermore, in the absence of integrated pharmacovigilance networks, there is often a lack of communication between local healthcare providers and national or global health authorities, limiting the ability to share and analyse data on drug safety.

To address this challenge, it is essential to develop low-cost, sustainable pharmacovigilance solutions tailored to the local context. For instance, mobile health (mHealth) technologies that utilise SMS-based systems or smartphone apps can be implemented to facilitate real-time reporting of ADRs by both patients and healthcare providers [45,46]. These tools can provide an efficient and affordable way to collect pharmacovigilance data, even in regions with limited infrastructure. Moreover, such solutions can be designed to operate offline or in low-bandwidth settings, ensuring that they are accessible in remote or underserved areas.

## Enhancing pharmacovigilance for mycetoma

To improve drug safety in mycetoma management, several key strategies must be adopted. These approaches include the following:

### Strengthening national pharmacovigilance programmes

Countries where mycetoma is endemic must prioritise the enhancement of their national pharmacovigilance programmes. Investing in healthcare infrastructure and providing adequate funding are the cornerstones of a robust pharmacovigilance system. This includes equipping rural health centers with the necessary tools to monitor adverse drug reactions, conducting routine liver, renal, adrenal and auditory function tests for patients on medical treatment and reporting findings to national health authorities [49].

Training healthcare professionals in pharmacovigilance practices is also essential. This could involve specialised courses or continuous education programmes that equip healthcare workers with the knowledge and skills to recognise, document, and report ADRs accurately. Proper training ensures that pharmacovigilance becomes an integral part of patient care rather than an afterthought. Additionally, national pharmacovigilance programmes should establish reporting databases that are integrated with global networks, such as the WHO’s global pharmacovigilance database (VigiBase) [50]. This will facilitate the collection of valuable data from mycetoma endemic regions, allowing for the early detection of trends and potential risks associated with long-term medical treatments.

In countries with limited resources, governments and health ministries should consider forming partnerships with international organisations, NGOs, and academic institutions to support the development of sustainable pharmacovigilance systems. These collaborations can help mitigate the financial and logistical challenges faced by resource-poor countries, enabling them to build more effective pharmacovigilance networks [51].

### Implementing mobile health (mHealth) solutions

Mobile health (mHealth) technologies offer tremendous potential for improving pharmacovigilance in mycetoma-endemic regions, particularly in low-resource settings where healthcare infrastructure may be inadequate. Mobile phones, which are widely available even in remote areas, can be used to bridge the gap between patients and healthcare systems [45,46].

Developing mobile applications or SMS-based systems that allow patients and healthcare professionals to report ADRs in real time could revolutionise pharmacovigilance efforts in mycetoma care. Such tools enable patients to communicate side effects directly with healthcare providers, facilitating faster interventions when adverse reactions occur. In addition, mHealth platforms can be designed to operate in offline or low-bandwidth environments, making them accessible in regions with limited internet connectivity. These platforms can also provide reminders for patients to take their medication, report side effects, or attend follow-up appointments, improving treatment adherence and fostering a more active role for patients in their care [45].

Moreover, mHealth solutions can be integrated into national pharmacovigilance systems, allowing the real-time data gathered from remote areas to be fed into centralised databases. This will help healthcare authorities to quickly identify and address safety concerns, contributing to data-driven policy decisions and more effective drug safety monitoring [44].

### Conducting more randomised clinical trials (RCTs)

One of the most pressing needs in mycetoma treatment is the lack of RCTs that thoroughly evaluate the safety and efficacy of both existing and novel therapies.. Many of the current treatments for mycetoma, such as itraconazole and ketoconazole, have been in use for years without sufficient evidence from large-scale clinical trials to support their long-term safety, particularly in the resource-limited settings where they are most needed. Only one clinical trial on mycetoma treatment was conducted and reported recently from Sudan [28]. Conducting more RCTs focused on mycetoma would help fill critical gaps in knowledge, particularly regarding the safety profiles of antifungal medications used in extended treatment regimens. These trials should also explore novel therapies, such as new drug formulations or combination treatments, and examine their safety and efficacy compared to traditional antifungals.

Pharmacovigilance systems must be integrated into these trials to collect real-time safety data. By embedding pharmacovigilance within clinical trials, researchers can monitor adverse events more effectively and make necessary adjustments to treatment protocols. This approach ensures that any new therapies brought to market are not only effective but also safe for widespread use in mycetoma patients. Moreover, the inclusion of pharmacogenomics in these trials could provide insights into how genetic variations affect drug metabolism and response, potentially leading to more personalised treatment strategies for mycetoma patients. Understanding these genetic differences could help minimise the risks of ADRs and improve treatment outcomes by tailoring therapies to individual patients’ genetic profiles [52].

### Expanding international collaboration and data sharing

Global collaboration is essential for improving pharmacovigilance in mycetoma management. Given the limited resources in many mycetoma-endemic regions, international cooperation can provide the necessary expertise, funding, and infrastructure to support national pharmacovigilance systems. Partnerships between governments, NGOs, research institutions, and pharmaceutical companies can help scale up pharmacovigilance efforts, ensuring that more patients are protected from adverse drug reactions. Furthermore, data sharing among countries and institutions can accelerate the detection of drug safety signals. The integration of national pharmacovigilance databases with global systems like VigiBase ensures that safety concerns identified in one region can be rapidly communicated to others, allowing for a coordinated response to potential risks.

**Mycetoma Research Center Experience with Pharmacovigilance and Drug Safety**

Since its founding in 1991, the Mycetoma Research Center (MRC), a WHO- Collaborating center for mycetoma and skin-NTDs, has been a leader in patient care, community engagement, and innovative research. Over the years, the MRC has used various medications and implemented comprehensive monitoring procedures to manage their side effects and complications. The center treatment guidelines emphasise regular patient follow-ups, with clinic visits every five weeks for clinical evaluations and necessary laboratory and imaging tests [53]. To improve patient care and accessibility, the MRC has embraced remote care strategies. These include telemedicine, digital consultations, and remote monitoring in endemic villages, allowing the center to track patient progress and side effects from a distance [54,55]. This approach ensures continuous care while reducing the need for frequent hospital visits, especially benefiting patients in remote areas and ultimately leading to better treatment outcomes. In women of childbearing age, Itraconazole poses risks, as it is contraindicated during pregnancy unless absolutely necessary. There is limited data on its use in pregnant women, but animal studies have shown risks of maternal and fetal toxicity. Additionally, Itraconazole has been associated with breakthrough bleeding and unintended pregnancies [55,56]. As such, the MRC requires a negative pregnancy test before starting treatment, and contraceptives are prescribed under gynaecological supervision. Some female patients have reported bleeding or miscarriage, with varying adherence to contraceptive regimens. Children, accounting for 30% of the MRC's patient population, require special attention due to the uncertainty around proper dosing and potential drug side effects, making close monitoring essential. Additionally, challenges with medication adherence and compliance are well-known issues. The MRC has developed a range of health educational materials, both written and digital, on mycetoma treatment and the importance of medication adherence. These resources, created in simple, easy-to-understand language and enhanced with illustrations, are distributed to patients and communities to raise awareness about mycetoma treatments [54]. Among the medications used at the MRC, amphotericin B, a systemic antifungal known for nephrotoxicity, was administered to four patients. One experienced reversible acute renal failure, and the treatment outcomes in the other patients were poor; its use was discontinued (unported data). At the MRC, ketoconazole was once widely used in eumycetoma treatment but was discontinued due to severe side effects, including hypersensitivity reactions, skin hyperpigmentation, adrenal insufficiency, hepatotoxicity, lips dryness and ulceration even before the FDA banned its use in 2013 [18,21]. Currently, Itraconazole is the first-line treatment for eumycetoma at the MRC. It is administered at 200 mg twice daily, but its known side effects, including hepatic and adrenal toxicity, require mandatory liver function tests every six weeks. Patients are also advised on dietary restrictions, as milk and dairy products, common diets for many, as it can reduce the drug's absorption. Amikacin sulphate, previously a first-line treatment for actinomycetoma, is now a second-line therapy due to its nephrotoxic and ototoxic effects [25]. It is reserved for patients who do not respond to the first-line medical treatment or present with severe disease [53]. Patients on Amikacin undergo close monitoring, and the drug is administered in cycles. Hence, at the MRC, clinical monitoring of adverse drug reactions, conducting routine liver, renal, adrenal and auditory function tests for patients on medical treatment and reporting findings to national health authorities are mandatory. To enhance the safety of drug use and improve patient care, the MRC partnered with the Sudan National Medicines and Poisons Board (NMPB), the main medicines regulatory body, to organise workshops on pharmacovigilance. The NMPB, as the Uppsala Monitoring Center (UMC) focal point in Sudan, provided training to MRC healthcare workers on detecting, identifying, and reporting adverse events, including rare and serious ones. This initiative increased healthcare professionals engagement in pharmacovigilance, contributing to patient safety and aligning with the United Nations Sustainable Development Goals (SDGs) 3, 4, and 17, focusing on good health and well-being, quality education, and partnerships for the goals [56]. 

**Role of clinical pharmacists in mycetoma management**

Clinical pharmacists play a crucial role in the management of mycetoma, from diagnosis and treatment to pharmacovigilance and community engagement. Clinical pharmacists, as accessible healthcare professionals in resource-limited environments, are uniquely equipped to enhance the comprehensive care of patients with mycetoma. They are crucial in the holistic management of mycetoma, offering vital services that include medication management, pharmacovigilance, community engagement, and patient education. Their participation guarantees that patients obtain safe, effective, and accessible treatment, thereby enhancing outcomes for this neglected disease.

## Conclusion

Pharmacovigilance is crucial for the safe and effective management of mycetoma, a condition that requires prolonged treatment with antifungal and antibiotic medications. Due to the chronic nature of the disease, patients often face extended drug exposure, increasing the risk of adverse drug reactions and drug interactions, particularly in regions where they may also be treated for other conditions. Early detection and management of ADRs through pharmacovigilance allow healthcare providers to adjust treatments, improving safety and adherence. Strengthening pharmacovigilance in mycetoma-endemic areas involves investing in healthcare infrastructure, providing education and training for healthcare workers, and leveraging mobile health (mHealth) technologies for real-time ADR reporting. These efforts are essential to ensuring patients receive the safest and most effective care, ultimately improving outcomes for those affected by this neglected tropical disease.
